# A randomized 3x3 crossover study to evaluate the effect of Hass avocado intake on post-ingestive satiety, glucose and insulin levels, and subsequent energy intake in overweight adults

**DOI:** 10.1186/1475-2891-12-155

**Published:** 2013-11-27

**Authors:** Michelle Wien, Ella Haddad, Keiji Oda, Joan Sabaté

**Affiliations:** 1 Department of Nutrition, School of Public Health, Loma Linda University, 24951 N. Circle Dr., Nichol Hall 1102, Loma Linda, CA 92350, USA; 2 Department of Epidemiology and Biostatistics, Loma Linda University, Loma Linda, CA, USA

**Keywords:** Avocado, Satiety, Overweight, Glucose, Insulin, Visual analog scale, Dietary compensation

## Abstract

**Background:**

The behavioral outcome of food ingestion is a complex process that involves psychological and biological factors. Avocados are nutrient dense with properties that may favorably impact energy balance. This study sought to evaluate if incorporating approximately one half of a Hass avocado by addition or inclusion into a lunch meal will influence post-ingestive satiety, glucose and insulin response, and subsequent energy intake among overweight adults.

**Methods:**

This was a randomized 3x3 single-blind crossover design study with 26 healthy overweight adults (mean ±SD age 40.8±11.0 years and BMI 28.1±2.4 kg/m^2^). Participants consumed a standardized breakfast followed by 1 of 3 lunch test meals [Control (C), avocado-free; Avocado Inclusive (AI); and, Avocado Added (AA)]. Participants rated five appetite sensations using a visual analog scale (VAS) before lunch and at specific intervals over 5 hours following the start of the test meal. Blood glucose and insulin were measured before lunch and at specific intervals over 3 hours following the start of the test meal. Mixed models were used to compare differences among the 3 test meals, and the area under the curve (AUC_0-xh_) was computed for the VAS and biological measures.

**Results:**

There were significant differences in the AUC_(0-5h)_ for the self-reported feelings of satisfaction (P=0.04) and desire to eat (P=0.05) in the mixed model analysis. Compared to the C test meal, the AA test meal increased satisfaction by 23% (P=0.05) and decreased the desire to eat by 28% (P=0.04) for the AUC_(0-5h)_. For the AUC_(0-3h)_, the AA test meal increased satisfaction by 26% (P=0.02) and decreased the desire to eat by 40% (P=0.01) as compared to the C test meal. Compared to the AI meal, the AUC_(0-3h)_ for blood insulin was higher in the C and AA meals (P=0.04 and P=0.05, respectively).

**Conclusions:**

The addition of approximately one half of a Hass avocado at a lunch meal can influence post-ingestive satiety over a subsequent 3 and 5 hour period in overweight adults. A caveat to these findings is that the avocado contained an additional 112 kcal, which may have accounted for the observed increase in satisfaction and decreased desire to eat. Future trials are warranted to evaluate the effects of avocado intake on weight management in adults of varying BMIs and among insulin resistant individuals.

## Background

The behavioral outcome of food ingestion is a complex process that involves psychological and biological factors that culminates in an individual’s overall 24-hour energy intake [1]. One of the components of the appetite system is satiety, which reflects a process that leads to increased fullness after a meal, a decline in hunger, and inhibition of further eating in the postprandial period.

In addition to sensory quality, the macronutrient composition, physical structure and energy density of a whole food may contribute to the modulation of satiety. More recent research has shown that the volume of a meal also influences satiety [2,3]. Additionally, the quality of the fat composition, i.e. degree of saturation of fatty acids in a food, may influence rates of oxidation and thermogenesis in animals and humans [4,5]. Furthermore, a single food may favorably impact energy balance according to its ability to offset spontaneous energy reduction at the next meal(s), which is known as the dietary compensation score [6].

The fresh pulp weight of Hass avocados is 72% water and contains only 1.7 kcal/g, therefore they are classified as a medium energy dense food (defined as a range between 1.5-4.0 kcal/g) [7]. Hence, when added to a meal they will increase the volume similar to other fruits and vegetables, which are food categories that have been previously shown to have a beneficial effect on weight control [8]. Further, the typical serving size is approximately one half of a medium size avocado (70 g) [9], which translates to being an excellent source of dietary fiber (5 g). Fiber is another food component strongly linked to enhancement of satiety [10] and modulation of the glucose and insulin responses to meals [11].

The connection between potential biological markers of appetite regulation continues to be an active area of research in normal weight and overweight populations. A 2007 meta-analysis by Flint et al. [12] has shown that the biological signaling of satiety by insulin in the overweight population is blunted, which could lead to the deleterious consequence of overeating at subsequent *ad libitum* meals and snacks. In light of the increased prevalence in overweight in humans and cross-sectional evidence showing an increase in snacking and total energy intake in the United States over the past three decades [13], the addition of approximately one half of an avocado at a specific meal(s) may be a simple dietary intervention to consider for individuals that consume excessive energy during specific snack and/or meal times. The aims of the present study are to evaluate if incorporating ~1/2 of a fresh Hass avocado by addition or inclusion into a lunch meal will influence post-ingestive satiety, the glycemic and insulin response, and subsequent energy intake in overweight adults.

## Methods

We conducted a randomized 3x3 single-blind crossover design study (three 1-day study periods scheduled 1 week apart) at Loma Linda University, Loma Linda, California. Using a within subject repeated measures design, we evaluated the effect of avocado intake on the short-term regulation of food by employing the use of one of three lunch test meals within a single day on three different days. Each participant received all treatments on the same day of the week and had a 1 week washout period between treatments.

### Eligibility criteria

Healthy overweight and moderately obese adults were recruited through the use of posters, flyers, and newspaper advertisements on the Loma Linda University campus and in the surrounding communities. A study web page was developed with a complete description of the study and online application form. Applications were also taken by phone.

Eligibility criteria were: age 25–65 years, body mass index (BMI, kg/m^2^) ≥25 and ≤35, weight stable for at least 6 months, normoglycemic, normotensive, sedentary or low level of habitual activity (less than 10 hours of exercise per week), non-smoker, not dependent on caffeine, and not taking any medications known to influence postprandial glucose and insulin levels. The recruitment process yielded 80 applicants and 56 individuals met the eligibility criteria. Forty-seven individuals attended information meetings and 30 were selected, plus 2 alternates. Four of the selected applicants declined participation due to unforeseen scheduling conflicts. Both alternates were included to achieve the targeted accrual of 28 participants and goal of 25 completers for adequate power (see Statistical Methods). One participant withdrew on the first day and one participant was asked to leave the second week due to non-compliance with the study protocol. The study was approved by the Loma Linda University Institutional Review Board and informed written consent was obtained from all participants.

### Anthropometric measurements

Height was measured to the nearest centimeter using a stadiometer on the first study day. Weight was measured using an internally calibrated segmental body composition scale/analyzer (model TBF-300A, Tanita®, Arlington Heights, IL) and recorded to the nearest 0.01 pound. BMI was calculated as weight(kg)/height(m^2^). The daily energy needs for each participant were estimated using the Harris-Benedict equation after adjustment for overweight status, which was subsequently multiplied by an activity factor of 1.3 for sedentary lifestyle. Participants were then assigned to receive either a 1600, 2000 or 2400 kcal daily meal plan.

### Study meals

Under the direct supervision of trained study personnel in the Loma Linda University Department of Nutrition Metabolic Kitchen, participants consumed the same standardized breakfast meal containing 25% of their estimated daily energy needs on each of the 3 assigned study days. For lunch, participants consumed 1 of 3 test meals: Control (C), avocado-free; Avocado Inclusive (AI); or, Avocado Added (AA) (see below). The standardized breakfast and the lunch test meals were designed to deliver the recommended levels of macronutrients according to the Acceptable Macronutrient Distribution Ranges developed by the Food and Nutrition Board of the Institute of Medicine [14]. The participants consumed 13-14% energy from protein, 49-51% energy from carbohydrate, and 35-38% energy from fat at the three lunch test meals. Further, the C and AI lunch meals delivered 35% of the participant’s daily energy needs and the AA lunch meal provided 41% of the daily energy needs (Table 1). All foods were precisely measured or weighed to the nearest gram using a digital scale and the meals were matched for taste and appearance. Participants were permitted to drink water with and between meals on the three study days according to their typical pattern of water intake.

**Table 1:** Percent of daily energy and macronutrient content of the 3 lunch test meals

	**Control**	**Avocado Inclusive**	**Avocado Added**
Energy, %	35	35	41
Carbohydrate, %	51	50	49
Protein, %	14	14	13
Fat, %	35	36	38

The standardized breakfast meal included orange juice, cornflakes, milk and a commercially prepared scone. The C test meal included a salad (mixed greens, cherry tomatoes, reduced fat Swiss cheese, Italian salad dressing), a refined grain French baguette and commercial chocolate chip cookies. Fresh, ripe Hass avocados (provided by the Hass Avocado Board) were sliced and included or added to the C test meal to produce the AI and AA test meals, respectively. The amount of avocado varied (range of ~50 to 90 g) with the energy needs of the participant [75 g (~1/2 of an avocado) for the 2000 kcal meal plan]. The portion sizes of the salad dressing and cookies were reduced in the AI test meal to match the energy and macronutrient content of the C test meal (Table 1).

The dinner buffet meal on the 3 study days was served 5 hours from the start of the lunch test meal and contained a variety of foods with pre-identified portion weight, macronutrient and calorie content. Participants were allowed to consume sweet and savory food options that differed in energy density in an *ad libitum* manner to allow for the assessment of postprandial food intake and dietary compensation. The number of portions of food items selected and consumed by the participants was directly observed and written in a discreet manner by a senior investigator and trained research staff. To supplement the written documentation of the food items taken and leftovers remaining on the plate, a hidden video camera was utilized to record the foodservice delivery process. The leftovers were photographed using a digital camera and weighed to the nearest gram using a digital scale. Two research assistants separately compared the written documentation with the videotape recording, still photos and weight of leftovers to produce a record of food intake for each participant. If any discrepancy existed between the two researcher’s records, a senior investigator reviewed all of the data sources to determine the most valid measurement of dietary intake.

Pre-portioned evening snacks were provided to participants at the conclusion of the *ad libitum* dinner meal and participants were asked to record any snacks consumed after leaving the research kitchen until going to bed or until midnight. Participants were contacted by phone the following morning by study personnel for a self-report of the intake of pre-portioned evening snacks. The energy and macronutrient intake subsequent to the lunch test meals was assessed based on the observed food consumed at the *ad libitum* dinner meal and from the participant’s self-reported consumption of pre-portioned evening snacks.

### Visual analog scales

By means of a mark on 100 mm line visual analog scales (VAS), participants rated their appetite sensations (hunger, fullness, satisfied, desire for a meal, and prospective food consumption). The VAS was completed before lunch and at approximately 30, 60, 90, 120, 180 and 300 minutes following the lunch test meal on each study day. The five scales were anchored at the low end with the most negative feelings (e.g. not at all) and opposing terms at the high end (e.g. extremely high).

### Sample collection and laboratory assessment

On the 3 assigned study days, participants arrived in the morning to the Nutrition Research Laboratory (NRL) after a 12-hour overnight fast for a baseline blood draw to measure glucose and insulin concentrations. Participants were free to engage in their normal morning routines but were instructed to return to the NRL by noon. The lunch test meal (C, AI or AA) was ingested within a 30 minute time period and additional blood samples were taken at approximately 30, 60, 90, 120 and 180 minutes following the start of the lunch test meal.

Venous blood samples were drawn and collected into vacutainer tubes (Becton Dickinson, Franklin Lakes, NJ) and centrifuged at 1500 x g at 4°C for 10 min. Serum and plasma were separated, aliquoted and frozen at −80°C until analyzed. Serum glucose was assayed with the glucose-oxidase-peroxidase enzymatic assay using kits supplied by Cayman Chemical (Ann Arbor, MI). Serum insulin was assayed using ELISA kits supplied by ALPCO Diagnostics (Salem, NH).

### Statistical methods

Sample size, power calculations and statistical analysis were performed utilizing SAS version 9.3 (SAS Institute, Cary, NC). All tests were two-sided and a value of *P* < 0.05 was considered significant. Under good experimental laboratory conditions, a sample size of 20 to 25 participants has been shown to be adequate to denote a 10% difference in AUC appetite ratings, which is considered to be a reasonable difference [15]. The target accrual was 28 participants to allow for a 10% dropout rate, which has been the observed dropout rate for our prior short-term feeding studies. A mixed model statistical approach was used to compare differences among the 3 test meals adjusting for study periods as fixed effects and treating participants as random effects. When significant findings were observed, Tukey post-hoc testing was performed to further elucidate the differences between the 3 test meals.

The weighted mean dietary intake of avocado was computed based on the number of participants assigned to each of the three aforementioned energy levels. The dietary compensation score across the C and AA test meals was calculated at the individual level using the following equation [6]: Percent Dietary Compensation = (Intake without load, C)-(Intake with load, AA)/Energy content of load x 100. More specifically, the dietary compensation at dinner was computed at the individual level by subtracting a subject’s dinner intake after the C lunch test meal minus the same subject’s dinner intake on the day of the AA lunch test meal, divided by the energy (or macronutrient) from the avocado consumed.

Two trained research assistants measured the VAS data to the nearest 0.1 cm and any discrepancy was resolved by a senior investigator. To compute the area under the curve (AUC) from zero to x hours (AUC_(0-xh)_), the minimum value of each subjectively reported VAS scale (in mm) over time was determined at the individual level and then the AUC above the minimum value was calculated using the linear trapezoidal rule. The AUC is reported as mm x minutes and was constructed by plotting the subjective values between 0 to 100 mm over time (minute) for each of the five VAS questions.

**Figure 1:**
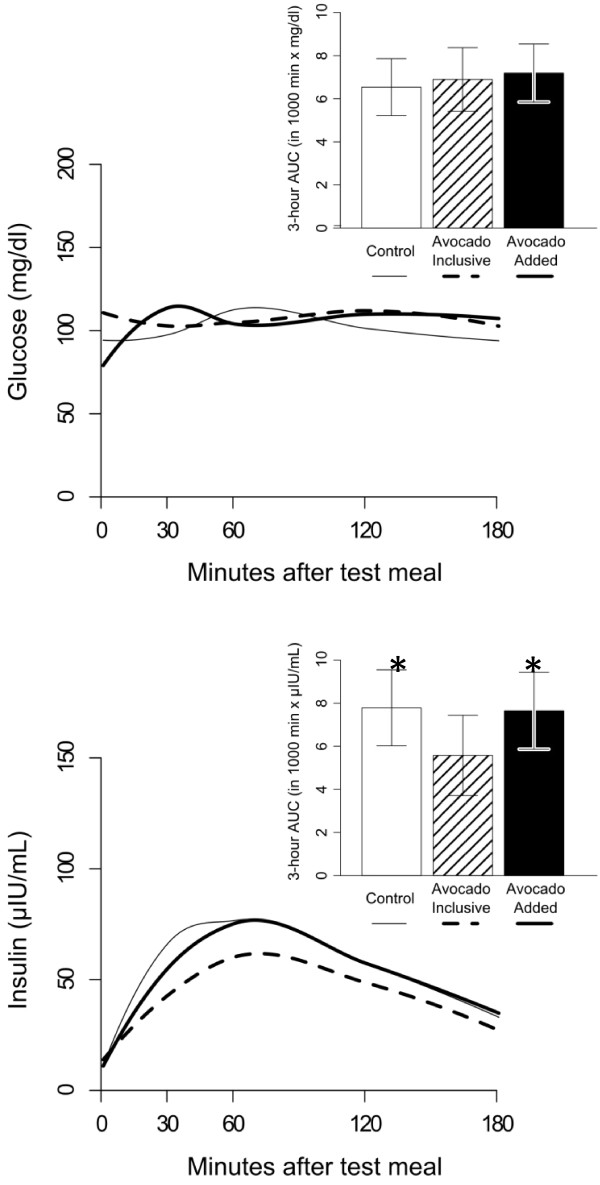
**Blood glucose and insulin levels after consumption of the 3 lunch test meals.** Three-hour area under the curve AUC_(0-3h)_ based on difference from baseline (time 0) is shown as an insert. Compared to the AI test meal, the blood insulin was higher in the C and AA test meals (P = 0.04 and P = 0.05, respectively).

**Figure 2:**
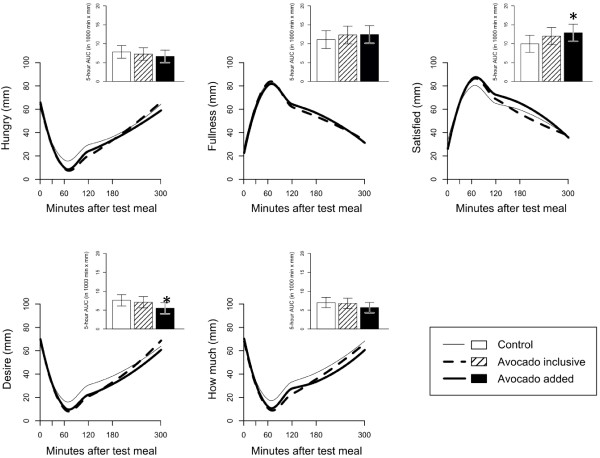
**Ratings for the five visual analog scale questions after consumption of the 3 lunch test meals.** Five-hour area under the curve AUC_(0-5h)_ based on difference from baseline (time 0) is shown as an insert. Compared to the C test meal, the AA test meal increased satisfaction by 23% (P = 0.05) and decreased the desire to eat by 28% (P = 0.04).

The VAS was completed and blood samples were scheduled to be taken at *approximately* 30, 60, 90, 120, and 180 minutes following the test meal. The exact time for each individual VAS and blood sample collection was recorded and these times were used for the analysis. Curved lines were generated to show the area under the curve for glucose and insulin (Figure 1) and the five VAS questions (Figure 2), which better represent the reality of the study data collection and analysis.

Data are expressed as adjusted mean ± SE unless otherwise noted.

## Results

The 26 participants that completed the study consisted of 16 women and 10 men with a mean ± SD age 40.8 ± 11.0 years and BMI 28.1 ± 2.4 kg/m^2^. Ten participants consumed the 1600 kcal meal plan, nine consumed the 2000 kcal meal plan and seven consumed the 2400 kcal meal plan. The weighted mean dietary intake of avocado was 67.5 grams, which contained 112 kcal, 1.3 g protein, 5.6 g carbohydrate and 10.4 g fat.

### Blood glucose and insulin changes

There were no significant differences between the 3 lunch test meals for AUC_(0-3h)_ blood glucose (Figure 1). Compared to the AI test meal, the AUC_(0-3h)_ for blood insulin was higher in the C and AA test meals (P = 0.04 and P = 0.05, respectively). Difference in blood insulin levels between treatments were observed at the 30 minute time point (P = 0.04) as follows: adjusted mean (95% CI) for C = 54μIU/ml (39, 74); AI = 34μIU/ml (25, 47); and, AA 42 μIU/ml (30, 57).

### Visual analog scale changes

There were significant differences in the AUC_(0-5h)_ for the self-reported subjective feelings of satisfaction (P = 0.04) and desire to eat (P = 0.05) in the mixed model analysis (Figure 2). Post-hoc testing revealed that compared to the C test meal, the AA test meal increased satisfaction by 23% (P = 0.05) and decreased the desire to eat by 28% (P = 0.04) for the AUC_(0-5h)_. For the AUC_(0-3h)_, the AA test meal increased satisfaction by 26% (P = 0.02) and decreased the desire to eat by 40% (P = 0.01) as compared to the C test meal (Table 2). Additionally, the AI test meal showed a tendency towards increasing satisfaction by 22% P = 0.07) as compared to the C test meal for the AUC_(0-3h)_. Lastly, the five measurements of appetite sensation tended to converge 5 hours after the lunch test meal.

**Table 2:** **Three-hour area under the curve AUC**^**a**^_**(0–3h) **_**based on difference from baseline (time 0) for the five visual analog scale questions between the 3 lunch test meals**

	**Control**		**Avocado Inclusive**			**Avocado Added**			
**Question**	**Mean**^**b**^	**SE**^**c**^	**Mean**^**b**^	**Difference**^**d**^	**P-value**^**e**^	**Mean**^**b**^	**Difference**^**d**^	**P-value**^**e**^	**P-value**^**f**^
How *hungry* are you?	3105	394	2358	−24%	0.30	2418	−22%	0.36	0.26
How strong is your feeling of *fullness*?	7249	771	8107	+11%	0.64	8189	+11%	0.58	0.55
How *satisfied* are you?	6340	726	8149	+22%	0.07	8562	+26%	0.02	0.02
How strong is your *desire* to eat?	2993	319	2263	−24%	0.16	1798	−40%	0.01	0.01
*How much* do you think you can eat?	2641	318	2185	−17%	0.45	2031	−23%	0.24	0.25

^a^The AUC is reported as mm x minutes and was constructed by plotting the subjective values between 0 to 100 mm over time (minute) for each of the five VAS questions.

^b^Adjusted mean from the mixed model analysis.

^c^Common standard error (SE) for all of the adjusted means.

^d^Percent difference compared to Control lunch test meal.

^e^P-value compared to Control lunch test meal.

^f^P-value for diet effect from the mixed model analysis.

### Intake at the ad libitum dinner meal and evening snack

Dietary intake at the subsequent *ad libitum* dinner meal and evening snack after each of the 3 lunch test meals was equivalent for total energy, macronutrients and percent energy from the macronutrients (Table 3). Compared to the C test meal, the percent dietary compensation for the AA test meal for energy, protein, carbohydrate and fat was 66%, 235%, 118% and 36%, respectively.

**Table 3:** Intake from the dinner meal and evening snack after the 3 lunch test meals

	**Control (C)**		**Avocado Inclusive (AI)**	**Avocado Added (AA)**	**P-value**^**c**^	**% Dietary Compensation**^**d**^	
	**Mean**^**a**^	**SE**^**b**^	**Mean**^**a**^	**Mean**^**a**^		**Mean**	**SE**
Energy (kcal)	1276	82	1193	1194	0.37	66	64
Protein, g (PRO)	42.6	2.8	39.2	39.0	0.27	235	204
Carbohydrate, g (CHO)	134.6	8.6	128.6	126.1	0.37	118	134
Fat, g	64.2	4.6	59.3	60.7	0.47	36	37
PRO, % total energy	13.4	0.3	13.2	13.0	0.36	-	
CHO, % total energy	42.8	1.1	43.5	42.6	0.72	-	
Fat, % total energy	44.7	1.1	44.3	45.4	0.58	-	

^a^Adjusted mean based on the mixed model analysis.

^b^Common standard error (SE) for all of the adjusted means.

^c^P-value for diet effect from the mixed model analysis.

^d^ $$\frac{\%\mathrm{Dietary}\;\mathrm{Compensation}=\left(\mathrm{Intake}\;\mathrm{without}\;\mathrm{load},C\right)–\left(\mathrm{Intake}\;\mathrm{with}\;\mathrm{load},\mathrm{AA}\right)\;\times \;100}{\mathrm{Energy}\;\mathrm{content}\;\mathrm{of}\;\mathrm{load}}$$.

[Note: The % dietary compensation at dinner was computed at the individual level by subtracting a subject’s dinner intake after the C lunch test meal minus the same subject’s dinner intake on the day of the AA lunch test meal, divided by the energy (or macronutrient) from the avocado consumed].

## Discussion

The results of this study suggest that the addition of ~ ½ of a Hass avocado at a lunch meal can influence post-ingestive satiety over a subsequent 3 hour and 5 hour period in overweight and moderately obese adults. Specifically, adding avocado to a lunch meal yielded a 23% increase in satisfaction (P = 0.05) and a 28% decreased desire to eat (P = 0.04) over a subsequent 5 hour period as compared to the avocado-free control lunch meal. Also, adding avocado to a lunch meal yielded a 26% increase in satisfaction (P = 0.02) and 40% decreased desire to eat (P = 0.01) as compared to the avocado-free control lunch meal over a 3 hour period. However, an additional 112 kcal was contained in the avocado, which may have accounted for the observed increased satisfaction and decreased desire to eat. Further, a 24% decreased desire to eat (P = 0.16) and 22% increase in satisfaction (P = 0.07) was observed over a 3 hour period after consumption of the isocaloric avocado inclusive lunch test meal as compared to the avocado-free control lunch meal. However, the changes in all five measurements of appetite sensation tended to taper off after 5 hours.

Energy intake at the subsequent *ad libitum* dinner meal and evening snack and dietary compensation did not differ between the 3 lunch test meals, which may have been due to the 5 hour time interval between the lunch test meal and *ad libitum* dinner meal. De Graaf and Hulshof [16] have previously reported that the weight or amount of food in a preload affects subsequent appetite and food intake for only up to two hours after the preload. These findings are consistent with the findings of equivalent energy intake at the subsequent dinner meal and evening snack in the current study, yet inconsistent with changes in two specific measures of appetite sensation that we observed at both 3 and 5 hours for the avocado added test meal. Further, Flint et al. [15] has reported that an 8-10% difference in the response magnitude relative to control in food intake or satiety score (AUC) is of practical relevance. We found differences of practical relevance for all five appetite sensation measurements between the C versus the AI and AA interventions ranging between 11-24% and 11-40%, respectively. However, we did not find a statistically significant difference for hunger, fullness or prospective food consumption between the 3 test meals.

Our overweight participants partially compensated for energy (66%) and fat (36%) intake and overcompensated for protein (235%) and carbohydrate (118%) at a subsequent *ad libitum* dinner meal and evening snack when avocado (weighted mean energy = 112 kcal) was added to the lunch meal. Thus, the majority of the energy provided by the addition of avocado to the diet was offset by dietary adjustments at the *ad libitum* dinner meal and evening snack. Others have reported that individual daily energy intake can vary by 20 to 30 percent, and that short-term dietary manipulations of less than ~400 kcal may not heavily influence dietary energy compensation [17,18], which may have been one of the reasons for the equivalent subsequent energy intake at the dinner meal and evening snack between the 3 study days.

There are two potential ways a whole food can be incorporated into a meal, addition or isocaloric replacement. Addition is when the food is simply added to a meal, which results in an increase in nutrients and total energy, whereas isocaloric replacement occurs when the food is included and other foods are simultaneously decreased or eliminated to compensate for the overall energy content of the meal. It is worth noting that the AUC_(0-3h)_ for blood glucose in the current study was equivalent between the 3 lunch test meals despite the additional mean energy (112 kcal) content and additional ~7 g carbohydrate in the AA lunch test meal. Avocados contain a unique seven carbon sugar (D-*manno*-heptulose) that does not contribute energy, and some believe it may support blood glucose control and weight management by reducing glycolysis via hexokinase inhibition [19]. Additionally, 30 minutes after the start of the lunch test meal the inclusion and addition of avocado significantly attenuated the rise in blood insulin levels by 37% and 22%, respectively (P = 0.04). Avocados are rich in antioxidants (e.g. polyphenolic compounds), which others have shown to be effective in improving insulin sensitivity in an overweight cohort [20]. Hence, including or adding avocado to a dietary pattern may assist in ameliorating the postprandial dysfunction in glucose homeostasis that may be present in overweight individuals.

The AUC_(0-3h)_ for blood insulin was lower in the AI test meal compared to both the C and the AA test meals, however this biological parameter did not significantly influence the five appetite sensation measurements between the AI and avocado-free C test meal (P = 0.07 to P = 0.64). It is worth noting that the five appetite sensation measurements for both the AI and AA test meals went in a favorable and similar direction, and borderline significant findings were found between the AI and C test meal in the context of increased satisfaction (P = 0.07) and a tendency existed towards reducing the desire to eat (P = 0.16).

Insulin and the incretin hormones covary in response to elevated postprandial glucose levels [21], which makes it challenging to uphold the glucostatic theory proposed by Mayer [22]. Andersen et al. have observed that postprandial levels of blood glucose are inversely associated with self-reported appetite and food intake [23], however others have shown no association between satiety and blood glucose levels using an intravenous carbohydrate infusion [24]. Thus, it is plausible that the incretin hormones were influenced by the fat and fiber from the addition of avocado to the AA test meal, which yielded an increase in satisfaction and a reduction in the desire to eat. Although fat delays gastric emptying, some studies have shown that protein in the diet has the most potent action on satiety followed by carbohydrate, and fat the least [25,26]. However, it is important to note that studies designed to evaluate the satiety level of fat usually add fat to a meal in the form of oil or shortening, which increases the energy density of the meal without appreciably altering the volume of the meal. Thus, the low satiating effect of fat found in some studies may have been mediated exclusively by the increase in energy density.

It is also worth noting that the intake at the *ad libitum* dinner and evening snack was similar between the AI and AA lunch test meals, and that the inclusion of avocado at a meal along with a concurrent reduction in other foods containing similar macronutrients favorably reduced the subsequent energy intake by 83 kcal (6.5%), and reduced the protein, carbohydrate and fat intake by 3.4 g, 6.0 g and 4.9 g, respectively. Avocados are a rich source of monounsaturated fatty acids, which are preferentially oxidized and increase thermogenesis as compared to polyunsaturated and saturated fatty acids. Thus, the inclusion of avocados to a dietary meal pattern may have additional implications in weight management in an overweight population.

Although we found a significant reduction in insulin levels and favorable changes in two specific measures of appetite sensation for the AI and AA lunch test meals, respectively, we did not observe any behavioral change in dietary intake at the subsequent *ad libitum* dinner meal and evening snack between the 3 test meals. However, this latter null finding should not be over-interpreted as the data presented in this study are for 3 separate days (one week apart) and additional dietary energy compensation is plausible over several days and weeks [27].

This study had several strengths and limitations. Our controlled “laboratory” type setting had high internal validity due to the high degree of sensitivity and control over the dietary intervention and study outcome measures. An additional strength is that we analyzed the AUC appetite sensation data as opposed to a single time point because analysis of individual time points is not physiologically independent and is prone to type 1 errors. A study limitation is that we did not measure dietary intake in-between the 3 assigned study days. An additional study limitation is that we provided a wide variety of foods at our *ad libitum* dinner buffet meal, which is at variance with the typical eating pattern of most individuals and is likely to delay satiation and facilitate increased food intake [28]. Lastly, we may have placed the participants in an atypical environment by not providing food to them for 5 hours.

## Conclusions

This study showed that the addition of ~ ½ of a fresh Hass avocado to a lunch meal favorably increased satisfaction and reduced the desire to eat over a subsequent 3 hour and 5 hour period in an overweight and moderately obese adult population. When avocados were either added or included to a lunch meal, similarities in five measures of appetite sensation were found over a subsequent 3 hour period. Given that the peak satisfaction effect was found to be within 3 hours of the lunch test meal, subsequent studies should address the offering of snacks as this may be of importance to overweight and moderately obese adults that typically consume large snacks between meals. Therefore, the addition of ~ ½ of an avocado at a specific meal(s) may be a simple dietary intervention to consider for individuals that consume large snacks (e.g. excessive energy) between meals.

Both the inclusion and addition of avocado to a lunch meal attenuated the rise in postprandial blood insulin levels 30 minutes after the start of the lunch meal as compared to the avocado-free control lunch meal. Additionally, the inclusion of avocado to a lunch meal yielded a significant reduction in blood insulin levels over a 3 hour postprandial period. The attenuation in the rise of insulin in the avocado inclusive intervention is worthy of future exploration in persons with insulin resistance and type 2 diabetes mellitus to determine if avocado intake can favorably influence measures of glucose homeostasis. Lastly, a longer trial would be beneficial to evaluate the effects of daily avocado intake on measures of appetite sensation and weight management in free-living normal weight, overweight and obese adults.

## Competing interests

The authors have no conflicts of interest or competing interests.

## Authors’ contributions

EH and JS designed the study. EH, MW and JS coordinated the study. MW and EH were responsible for data collection, analysis and quality control. MW, EH, JS and KO were involved in the statistical analyses. All authors contributed to the interpretation of data. MW wrote the first draft of the manuscript and all authors critically reviewed and revised the manuscript. JS obtained the funding for the study. All authors read and approved the final manuscript.
